# Prevalence and determinants of frailty in the absence of disability among older population: a cross sectional study from rural communities in Nepal

**DOI:** 10.1186/s12877-019-1290-0

**Published:** 2019-10-22

**Authors:** Uday Narayan Yadav, Man Kumar Tamang, Tarka Bahadur Thapa, Hassan Hosseinzadeh, Mark Fort Harris, Krishna Kumar Yadav

**Affiliations:** 1 Centre for Primary Health Care and Equity, UNSW, Sydney, Australia; 2 Forum for Health Research and Development, Dharan, Nepal; 3 Queensland Brain Institute, The University of Queensland, Brisbane, Australia; 4 University of Wollongong, Wollongong, Australia

**Keywords:** Depression, Frailty, Older, Prevalence, Primary care

## Abstract

**Background:**

Longevity and frailty have significant implications for healthcare delivery. They increase demands for healthcare service and surge risk of hospitalization. Despite gaining global attention, determinants of frailty have remained unmeasured in the rural community settings in Nepal. This study aimed to address this gap by accessing the prevalence and determinants of frailty in the absence of disability among older population living in rural communities in eastern Nepal.

**Methods:**

We conducted a cross-sectional analytical study of 794 older adults aged ≥60 living in the rural part of Sunsari and Morang district of eastern Nepal between January and April in 2018. Multi-stage cluster sampling was applied to recruit the study participants. Study measures included socio-demographics; Frail Non-disabled scale (FiND) measuring frailty, Barthel’s Index measuring basic activities of daily living and Geriatric depression scale. Determinants of frailty in the absence of disability were identified using generalized estimating equation (GEE).

**Results:**

About 65% of the participants self-reported the presence of frailty in the absence of disability. In the adjusted models, those from underprivileged ethnic groups, lack of daily physical exercise, presence of depressive symptoms and those not getting enough social support from family were found to be significantly associated with frailty among older participants.

**Conclusions:**

The prevalence of frailty in the absence of disability was high among rural community old population living in eastern Nepal. Our findings suggest that need of frailty awareness (both for clinicians and general public), so as to avoid negative consequences. To reduce the healthcare burden early screening frailty in primary care has potentials to prevent implications of frailty in Nepal.

## Background

Longer life is valuable, and existing evidence suggests that that older people today are experiencing better health compared to previous generation [1]. However, physical and mental capacities are often decreased by increasing aging [2].

Nepal, a low-income country like other countries has achieved significant progress in reducing the infant and premature mortality [3]. This has led to better life expectancy for both women [71.88 years] and men [68.66 years] in 2016 [4]. Now healthy aging is an emerging challenge for public health in Nepal [5].

Frailty is a common geriatric syndrome which is characterized by age-associated declines in physiologic reserve and function, leading to increased vulnerability, ranging from adverse health outcomes (falls, disability, and institutionalization) to death [6, 7]. Epidemiological studies on frailty mostly conducted in high-income countries [9, 10], have associated frailty with depression, malnutrition, polypharmacy, poor hearing, lack of exercise, poor family support, presence of co-morbidities and poorer self-reported health status [8, 11–15]. A study from Nepal conducted among 253 beneficiaries of Gurkha Welfare Trust, a welfare centre using a clinical frailty scale reported 46.2% of frailty among participants aged ≥60 years old [16].

Epidemiological transitions show the increase in the number of older populations in Nepal [4]. Available data suggest that the quality of life of older population is undermined by changes in traditional family structure, modernization, lack of career and the presence of long-term disease [4] This can cumulatively increase the risk of frailty. Despite this, there is scarceness of evidence on the frailty in the absence of disability among older population in the community setting of rural Nepal. This study is an attempt to address this gap by exploring prevalence and determinants of frailty in the absence of disability among older population in the rural part of Sunsari and Morang district of eastern Nepal.

## Methods

### Study designs and participants

This was a community-based cross-sectional study conducted among older people adults aged ≥60 years old living in the Morang and Sunsari districts of Nepal, carried out between January and April in 2018. A multi-stage cluster sampling approach was used to select study subjects. The sample size of 847 was calculated based on following assumptions: prevalence = 50%, sampling error = 5.0%, CI = 95.0%, design effect = 2 and non-response rate = 5.0%. A total of 794 of the sampled eligible participants responded to the study. In the first stage, four Rural Municipalities (RMs) were randomly selected from the list of RMs within each Morang and Sunsari District. Second, five wards were randomly selected in each of the selected RMs. Table 1 presents the details of sampling strategy. Finally, individuals were selected randomly from the list of eligible subjects provided by the RMs representative and were interviewed by the trained interviewers in the community setting.

**Table 1:** Sampling details of this study

	# of older people in each randomly selected ward of RM	Number of participants selected from each selected ward
Sunsari District		
Rural Municipality 1		
Ward a	640	18
Ward b	702	20
Ward c	806	23
Ward d	774	22
Ward e	543	17
Rural Municipality 2		
Ward a	663	19
Ward b	636	18
Ward c	723	21
Ward d	601	18
Ward e	627	19
Rural Municipality 3		
Ward a	1004	29
Ward b	732	22
Ward c	888	26
Ward d	844	24
Ward e	592	17
Rural Municipality 4		
Ward a	784	23
Ward b	683	20
Ward c	709	21
Ward d	814	24
Ward e	684	20
Morang District		
Rural Municipality1		
Ward a	640	20
Ward b	702	20
Ward c	806	23
Ward d	774	22
Ward e	722	21
Rural Municipality2		
Ward a	936	27
Ward b	804	23
Ward c	601	17
Ward d	508	16
Ward e	65	19
Rural Municipality 3		
Ward a	832	24
Ward b	701	20
Ward c	824	24
Ward d	502	15
Ward e	802	23
Rural Municipality 4		
Ward a	784	23
Ward b	683	20
Ward c	709	22
Ward d	814	24
Ward e	789	23

Proportionate sampling was adopted to reach the sample size in each rural mucipality of the respective districts

The eligible study population included adults aged ≥60 years old who had lived in the community for the past year and were Nepali nationals and willing to complete study survey. Informed consent was obtained from all the study participants (thumb impressions from those who were not able to read and write) and was informed about the right to free to withdraw or opt out at any point without any penalty. The exclusion criteria included residing in nursing care, being mentally disabled (clinically proved schizophrenia, bipolar mood disorder), being seriously ill (terminal illness like cancer, chronic kidney disease), having a hearing disability or being unable to communicate. The study protocol was approved by the Ethics Committee of Nepal Health Research Council, Ministry of Health.

### Data collection and study variables

The study used both semi-structure interviews and validated survey instrument to collect data.

### Primary measurement

Frailty was the primary outcome, which was measured using “Frail non-Disabled” (FiND) questionnaire [17], the novel instrument designed to measure frailty syndrome and disability in community-dwelling older persons.

The FiND questionnaire (α = 0.82) contains five sections ranging from A to E. Items A and B measure mobility disability whereas item C measures weight loss; item D measures exhaustion; and item E measures level of physical activity. Participants were categorized “disabled” if item A + B ≥ 1, “frail” if A + B = 0 and C + D + E ≥ 1 and “robust” if A + B + C + D + E = 0.

### Independent variable measurement

Independent variable included age group; gender; ethnicity; religion; marital status; living arrangement; literacy status; occupation; monthly personal income; smoking habit; alcohol drinking habit; tobacco chewing habit; physical activity; presence of any co-morbidities; depressive symptoms; activities of daily living; ; and getting enough support from family members/caregivers. Measurement of concentration problems included “failing to recall the position of objects and forgetting to perform activities in time by the older people if instructed by family members or caregivers in the last 30 days”.

These co-variates are described in the published paper authored by Yadav et.al [18]. Barthel’s scale measuring activities of daily living [19] was used to assess daily living activities. Depressive symptoms were assessed using a short version of the geriatric depression scale [20].

The English version of the survey was first translated to Nepali and then translated (forward-backward translation) back to English by two researchers to check the consistency. A small community level workshop was conducted to arrive to final version of the tool by considering the remarks from older population.

### Statistical analyses

The statistical analyses were performed using the Statistical Package for Social Sciences (SPSS 23.00). Normality of the data was assessed using both visually (histogram with normal curve) and normality test (Shapiro-Wilk test). An association between the categorical variables was checked using Chi-square test. Variables that were significantly associated (*p*-value ≤0.05) with the outcome variables in univariate analysis were considered in the stepwise multivariable analysis. The generalized estimating equation (GEE) was used to identify the factors associated with frailty in absence of disability among the older population.

## Results

### Study sample characteristics

Complete data were collected from 794 study participants. The mean age of the respondents was 70.16 (± 8.54) years old for male and 69.70 years old (± 8.86) for females. Table 2 shows the characteristics of the study population. Socio-demographic findings showed that majority of the study participants were in the age group of 60–69 years (55%), were from Brahmin/Chhetri/Thakuri ethnic group (38%), 79% were ascribed to Hindu religion, married (53%), living with family members (73%), illiterate (80%), unemployed (53%) and had no personal monthly income (66%). Majority of the study participants had a smoking and tobacco chewing history. Only 22.92% were ever involved in physical exercise. The variables measuring health status showed that 61.58% of the study participants had at least two chronic condition (combination of hypertension, cardiovascular disease, arthritis, respiratory disease etc) and more than half (55%) had depressive symptoms. About 47% needed assistance in daily living activities and 39% had memory concentration problems in past 30 days prior to a survey.

**Table 2:** Participants’ characteristics (*n* = 794)

Variables	Presence of non-disabled frailty		*P*-value
	Yes	No	
Age group (years)			
60–69	315 (61.0)	125 (45.0)	0.000
70–79	146 (28.3)	89 (32.0)	
≥ 80	55 (10.7)	64 (23.0)	
Gender			
Male	268 (51.9)	132 (47.5)	.13
Female	248 (48.1)	146 (52.5)	
Ethnicity			
Brahmin/chhetri/thakuri	41 (7.9)	28 (10.1)	
Aadiwasi/Janajatis	175 (33.9)	123 (44.2)	0.000
Dalit	104 (20.2)	53 (19.1)	
Madheshi	169 (32.8)	74 (26.6)	
Other ethnical groups	27 (5.2)	0 (0)	
Religion			
Hinduism	403 (78.1)	222 (79.9)	
Buddhism	8 (1.6)	11 (4.0)	.03
Islam	91 (17.6)	34 (12.2)	
Christianity	14 (2.6)	11 (4.0)	
Marital status			
Married	292 (56.6)	133 (47.8)	.04
Widow/widower/divorced/separated/unmarried	224 (43.4)	145 (52.2)	
Living arrangement			
Stays with family including spouse	389 (75.4)	194 (69.8)	.21
Stays with spouse only	88 (17.1)	60 (21.6)	
Stays alone	39 (7.6)	24 (8.6)	
Literacy status			
Illiterate	398 (77.1)	238 (85.6)	.005
Literate	118 (22.9)	40 (14.4)	
Occupation			
Employed	248 (48.1)	116 (41.7)	
Unemployed	259 (50.2)	159 (57.2)	.15
Retired/Pensioner	9 (1.7)	3 (1.1)	
Monthly personal income (NPR)			
No income< NRs.500	340 (64.9)	184 (35.)	.007
NRs.500–2000	79 (56.4)	61 (43.6)	
>NRs. 2000	97 (74.6)	33 (25.4)	
Smoking habit			
Never smoker	194 (37.6)	106 (38.1)	.94
Having smoking history	322 (62.4)	172 (61.9)	
Alcohol drinking habit			
Never drinker	321 (62.2)	183 (65.8)	.35
Having alcohol drinking history	195 (37.8)	95 (34.2)	
Tobacco chewing habit			
Never tobacco chewer	256 (49.6)	155 (55.8)	.87
Having tobacco chewing history	260 (50.4)	123 (44.2)	
Physical activity			
Daily physical exercise	97 (18.8)	85 (30.6)	.000
No physical exercise at all	419 (81.2)	193 (69.4)	
Presence of any co-morbidities			
Yes	296 (57.4)	193 (68.4)	.001
No	220 (42.6)	85 (30.6)	
Depressive symptoms’			
Yes	259 (50.2)	184 (66.2)	.000
No	257 (49.8)	94 (33.8)	
Activities of daily living			
Dependence	169 (32.8)	204 (73.4)	0.000
Independent	347 (67.2)	74 (26.6)	
Memory concentration problems in last 30 days			
Yes	169 (32.8)	139 (50.0	.000
No	347 (67.2)	139 (50.0)	
Getting enough support from family members/caregivers			
Yes	80	40	.07
No	436	238	

### Prevalence of frailty

The prevalence of frailty, disability and robustness were examined using of the FiND questionnaire. Of total 794 samples, 65% [CI: 61.68–68.32] were “frail” (non-disabled), 28% [24.88–31.12] were “disable” and only 7% [CI: 5.23–8.77] reported being robust (Table 3).

**Table 3:** Community-dwelling older persons with frailty syndrome with FiND screening tool (*n* = 794)

	Category	n	% [95% CI]
FiND	Robust	56	7.0 [5.23–8.77]
	Frail (non-disabled)	516	65.0 [61.68–68.32]
	Disabled	222	28.0 [24.88–31.12]

### Factors associated with frailty

Table 4 presents multivariable regression results indicating the factors independently associated with frailty. After adjusting for socio-demographic variables, being from any of three underprivileged ethnic groups [Indigenous: aOR = 1.07, CI: 1.01–1.14; Dalit: aOR = 1.13, CI: 1.03–1.14 and, Madhesi: aOR = 1.07, CI: 1.00–1.14], lack of daily physical exercise [aOR = 1.22, CI:1.15–1.30], presence of depressive symptoms [aOR = 1.06, CI: 1.02–1.10] and those not getting enough social support from family [aOR = 1.04, CI: 1.01–1.08] were significantly associated frailty.

**Table 4:** Factors independently associated with frailty in the absence of disability among rural communities’ old population

Variables	Univariate model OR [95% CI]	Multivariate model aOR[95% CI]
Marital status		
Married	1	1
Others Widow/widower/divorced/separated/unmarried	1.04 (1.00–1.09)	1.00(.96–1.04)
Ethnicity		
Brahmin/chhetri/thakuri	1	1
Indigenous group	1.16 (1.11–1.21)	1.07 (1.01–1.14)
Dalit	1.19 (1.09–1.31)	1.13 (1.03–1.14)
Madheshi	1.10 (1.04–1.16)	1.07 (1.00–1.14)
Other ethnical groups	.000	.000
Religion		
Hinduism	1.09 (1.06–1.12)	.98(.84–1.15)
Buddhist	1.46 (1.23–1.74)	.98(.84–1.14)
Islam	1.30 (1.11–1.51)	1.14(.91–1.42)
Christianity	1	1
Daily physical exercise		
Yes	1	1
No	1.29 (1.21–1.37)	1.22 (1.15–1.30)
Depressive symptoms		
No	1	1
Yes	1.08 (1.04–1.13)	1.06 (1.02–1.10)
Getting enough support from family members/caregivers		
Yes	1	1
No	1.08 (1.04–1.12)	1.04 (1.01–1.08)

## Discussion

The growing aging population increases the demand for and use of older adult(s)-friendly health services in Nepal [4]. The National Health Policy in 2014 aimed to deliver quality health services to the all of the citizens (Universal Health Coverage) and provide basic health services at free of cost [21] However, delivery of health services conducive to the aging population health like frailty is not envisaged well. In light of this, there is a yawning gap in the assessment of non-disabled frailty and its determinants among older adults in Nepal. This study aimed to determine the prevalence of frailty and its determinants among older adults living in the rural communities in Eastern Nepal. To the best of our knowledge, this is the first study to explore frailty in the absence of disability among the geriatric population residing in the community settings of Nepal.

Our finding shows that the prevalence of frailty was 65% among the older population. Frailty is a recognized health problem of older people health and this finding is in line with strong rationale highlighting the need of evidence of frailty in Low and Middle-Income Countries (LMICs) [22]. In line with our finding, Devkota et al., reported 46.2% of Nepalese pensioners had a degree of frailty [16]. The population previously studied are Nepalese origin ex-British army whereas our study involves the general Nepalese citizen of the community setting. Our estimate is higher than the estimates of frailty from a single multicounty study including China (13.1%), India (55.5%) and a nationally represented study from Singapore (5.7%) [23, 24]. This might be explained by the fact that these studies used different frailty instruments. Such discrepancies indicate that there is a need to develop an internationally standardized frailty assessment measurement detecting frailty among older populations. In our study setting, the high rate of morbidity may relate to “over reporting of health problems” to get better health care from the health service provided by government of Nepal. This indicates that health decision and policy makers should be aware of social desirability bias while designing any intervention aimed to improve healthy wellbeing or health ageing.

Our results showed that older people from underprivileged ethnic groups, those with depressive symptoms and not doing daily physical exercise, and those not getting enough social support from family were found to have frailty in the absence of disability in rural older population. Notably, we found increased odds of frailty among three underprivileged ethnic groups as compared to higher caste, which may be related to the poor socio-cultural and socio-economic class of the caregiver of the older people population. Caste /Ethnicity have been a central feature in Nepal to describe level of poverty, poor health literacy and health status [25]. The condition of underprivileged group is miserable in the rural setting as compared to higher caste as they do not have equitable access to the prevailing service because of multiple barriers [26]. This could be related to poor purchasing and consumption capacity for food, which might have affected their nutritional status and, therefore put older people at higher risk of frailty. Higher castes in Nepal have the highest per capita income as compared to lower castes [27]. In light with our findings, previous research has showed the linkage between socio-economic disadvantage with higher allostatic load “known as wear and tear” of the body, which in turn related to frailty [28, 29].

Among older adults who have no social support from family members progressive were frail compared to their counterparts. This can be explained by the fact; lack of social support makes the older people prey for psychological illness reflecting that vulnerability worsens their health status. The literatures support the fact that lack of social support makes the older people feel helpless and lonely, which might affect consumption of health food, poor appetite and poor adherence to the medicines [30]. In total, this affects the nutrition status of the older people. Theories of environmental gerontology state that people are influenced by an ongoing interchange between the individual and their physical and social environment [31]. Our findings are supported by the findings from study that revealed increased social support was associated with less-steep increases of frailty over time [32]. Similarly, Lurie et al. found an association between social support and lower frailty levels 10 to 13 years later among older adults less than 65 years of age [33].

Our finding in terms of depressive symptoms and frailty is in line with existing literature [12, 13]. Presence of depressive symptoms is often correlated with the increased risk of frailty [34]. In relation to physical activity, our finding showed that lack of physical exercises increases the odds of frailty by more than two times [35, 36]. The results from the population-based cohort study showed that physically active older adults as compared to their physically inactive counterparts found to be associated with lower all-cause mortality among the frail, pre-frail and robust individuals [37]. Similarly, the findings from multifactorial Frailty Intervention Trial (FIT) found that included balance, strength, and endurance exercise showed the lower prevalence on frailty and improved mobility among the intervention arm compared with the control group [38]. It is worth mentioning that physical exercises have positive impact on the individual functional life and improving health consequences of the frail people. Therefore, exercise could be a key modifiable intervention for improving physical function and in preventing and reducing the frailty.

Despite invaluable findings, this study has some limitations, which should be considered in any interpretation and generalization. The participants were from eight rural municipalities of Morang and Sunsari district, Nepal; thus, the results can be only generalized to the studied district with caution. Our findings relied on self-reported data, where social- desirability bias may have occurred. Moreover, the measurement of lifestyle factors like alcohol use, smoking and physical activity was not done using any standard instrument. Further, since this study was cross-sectional, causal relationships cannot be established. The notifiable strength of this study is: large sample size with response rate more than 90%, strong methodology and adoption of FiND questionnaire for the first time in Nepalese settings and, we recommend more studies using this tool.

## Conclusions

Noting the high prevalence in the community, we suggest the screening of frailty at primary health care could be a ground-breaking work to avoid the beginning of an irreversible disabling process. We also emphasize on frailty awareness on preventive aspects (both for clinicians and general public), so as to avoid negative consequences, to reduce the healthcare burden. Further, we recommend the need for longitudinal follow-up national-level studies on frailty among older population in Nepal. Additionally, our finding in particular suggest that community-based intervention having physical activity as essence component can be researched to see the promising outcomes suggested by the literatures of other settings.

The evidence generated in this study shows that non-disabled frailty is very common among the older population in rural communities in Nepal and evidence-based strategies are needed to address this growing public health challenge.

## Data Availability

The datasets used and/or analysed during the current study are available from the corresponding author on reasonable request.

## Abbreviations

aOR: Adjusted Odds Ration
CI: Confidence Interval
FiND: “Frail non-Disabled” questionnaire
GEE: Generalized estimating equation
RMs: Rural Municipalities
SPSS: Statistical Product and Service Solutions
